# Supplementary material for: MetaRibo-Seq measures translation in microbiomes
Source: Nat Commun. 2020 Jun 29;11:3268. doi: 10.1038/s41467-020-17081-z (PMC7324362; doi:10.1038/s41467-020-17081-z)
Supplement: Supplementary file 10 — Supplementary Data 7 [file 41467_2020_17081_MOESM10_ESM.zip › File2/Confidence_High_Taxonomy/91513_out.krona.html]

Javascript must be enabled to view this page.

members
magnitude
magnitudeUnassigned
count
unassigned
taxon
rank

91513\_out

5

5
superkingdom
2

5
phylum
1239

5
class
186801

186802
5
order

1
family
990719


SRS075984\_contig\_number\_contig-100\_31896.71431
1930012
species
1

31979
3
family

1485
genus
3


SRS1041140\_contig\_number\_16526SRS143895\_contig\_number\_20785SRS148196\_contig\_number\_contig-100\_3995.196612
species
3
1262781

family
1
541000

1
genus
292632

1
species
1897022

SRS020869\_contig\_number\_7996
